# How young people understand mental health survey questions: a qualitative think-aloud study from the Swedish National Public Health Survey

**DOI:** 10.1186/s12889-026-30215-0

**Published:** 2026-11-09

**Authors:** Ellinor Tengelin, Monica Bertilsson, Hrafnhildur Gunnarsdottir, Sofie Lundström

**Affiliations:** 1 Department for Health Sciences, Occupational Health Science, Mid-Sweden University, Ostersund, SE-831 25 Sweden; 2 Insurance Medicine, School of Public Health and Community Medicine, Institute of Medicine, University of Gothenburg, Gothenburg, SE-405 30 Sweden; 3 Public Health Agency of Sweden, Folkhälsomyndigheten, Solna, SE-171 82 Sweden; 4 Department for Health Sciences, University West, Trollhattan, SE-461 86 Sweden

**Keywords:** Anxiety, Mental health, Public health surveys, Stress, Think-aloud, Young people

## Abstract

**Background:**

In light of the diverging trends in young people’s mental health, where reports of declining health coexist with indicators of improved well-being, this study aimed to explore young people’s reasoning about survey questions concerning psychological distress in the Swedish public health survey.

**Methods:**

A ‘think-aloud’ design was employed, where 33 young people aged 16–25 were interviewed about public health survey questions concerning mental health. The interviews were analysed using qualitative content analysis.

**Results:**

The participants struggled with both the wording and the framing of the questions: the concepts of anxiety, worry, and unease were perceived as overlapping yet difficult to separate; stress was experienced as both positive and negative; time frames were interpreted in inconsistent ways; and response options were sometimes regarded as too narrow. These ambiguities indicate that responses were influenced by participants’ interpretations rather than solely by the phenomena the questions were intended to capture.

**Conclusion:**

Trends in self-reported mental health must be interpreted in light of how survey items are understood. While continuous review of public health surveys is necessary to maintain validity, this complicates long-term trend analyses. There is a risk that current survey data either over- or underestimate changes in mental health if younger generations understand and respond to survey items differently than earlier generations. These interpretations could persist as cohorts age, underscoring the need for future research on how different generations understand and respond to mental health survey questions across the life course. Survey data alone seem insufficient to capture key nuances of young people’s mental health, making qualitative approaches a necessary complement to public health policy and practice. Advancing this field also requires the development and validation of more contextually relevant survey questions that better reflect how contemporary youth understand and talk about mental health.

## Introduction

Population-based surveys provide a crucial foundation for public health action [1], and the way respondents interpret questions about their health has direct implications for how population health is monitored. Mental health problems have been recognised as being among the most significant health challenges facing young adults today [2], yet discrepancies in the reporting of mental health [3–5] raise concerns about how survey items are understood by young adults themselves.

The reporting of symptoms of depression and anxiety has increased among young adults over the last few decades [2], an observation that has been linked to global mega-trends such as economic insecurity, individualism, academic pressure, climate change, and social media use [6, 7]. In Sweden, reports of mental health among young people paint a somewhat contradictory picture [3–5]. National data indicate that most young people (78% of those aged 16–29) report high levels of mental well-being, although this is still the lowest proportion of any age group [8]. One recent Swedish study showed that although overall levels of self-reported depressive symptoms in adolescents aged 13–17 were similar in 2004 and 2019/20, the later cohort included more adolescents with very low symptom levels. At the same time, adolescents who reported psychological distress scored higher on measures of self-deprecation and negative thoughts [9]. Population-level data also suggest age-related patterns across a broader age range. In 2024, more than half of women and 35% of men aged 16–29 reported mild symptoms of unease, worry, or anxiety, and 37.1% of women and 21.1% of men in the same age group reported stress. This is indicative of a negative trend: since 2006, the proportion reporting anxiety, worry, or unease increased across all age groups, and the proportion reporting stress increased among those aged 16–29, from 17.7% to 28.8% [10, 11]. The proportion of young people aged 16–29 who report severe symptoms of unease, worry, or anxiety increased from 4.3% in 2006 to 9.6% in 2024 among boys and from 9% to 23.3% among girls [10]. In contrast, changes in other age groups were substantially smaller. Among women aged 30–44 years, the prevalence increased from 6.5% to 11.3%, whereas a decrease was observed in the oldest age group among both men (from 2.8% to 2.5%) and women (from 5.4% to 3.4%) [10]. Taken together, these findings do not indicate a single, uniform trajectory in young people’s mental health. They show that high levels of self-reported well-being coexist with increasing reports of symptoms in some groups, while other indicators remain stable or show lower symptom levels in some cohorts. The pattern also varies according to age, sex, symptom severity and the outcome measured.

The academic discourse over the past decade has engaged with how to interpret the reported increase in mental health problems. Hagquist [3] found that increases in self-reported mental health complaints in Swedish data from school-aged children were concentrated among older adolescents, especially girls, rather than across all age groups, and Bremberg [4] argued that Sweden experienced a more marked rise in internalising symptoms than comparable Nordic countries and the Netherlands. Eriksson & Stattin [5] caution against interpreting the increase in reported symptoms as evidence of a general decline in mental health, as they note that the proportion of adolescents with the poorest mental health has remained stable over time, while increases have mainly occurred in adolescents with less severe complaints. A similarly cautious reading is supported by Wikberg et al. [9], who found no significant increase in total depressive symptom scores across two Swedish cohorts, despite higher scores on some items, highlighting the need to interpret adolescent self-assessments in relation to broader societal change. Observations of young people’s mental health need to be interpreted with caution, acknowledging the impact of factors such as language, societal changes, and current trends in young people’s self-assessments [9].

Against this backdrop, survey questions become important, as they are the main tool through which these trends are documented. Many health surveys rely on self-assessments of health. While standardised survey questions offer an established method for monitoring populations, the way questions are formulated and understood plays a crucial role in ensuring that the survey accurately measures what it intends to measure [12, 13]. Self-reported symptoms are often assumed to be representative of ill-health, but respondents’ interpretations of what is asked about can vary greatly. For example, young people have been found to understand the same mental health symptoms asked for in surveys either as signs of ‘deep-seated problems’ or as reflections of ‘everyday challenges’ [14].

Surveys are not only measurement tools, but respondents may develop new insights or shift their understanding as they read, reflect on, and answer survey questions [15, 16]. Diagnostic categories structure the ways in which people make sense of their health and illness [17]; surveys similarly impose categorical frameworks that may guide respondents’ interpretations and responses. In addition, the societal discourse around mental health is evolving, with decreasing stigma and greater openness influencing how young people perceive experiences and whether they feel comfortable acknowledging and reporting them [18]. Understandings of mental health may be changing particularly rapidly among young people, who are influenced by rapid developments such as digitalisation and social media. Thus, a question about mild symptoms of anxiety might elicit very different responses today compared with times when such experiences were more stigmatised. These shifts call for continuous critical review of how public health surveys capture mental health.

### The Swedish setting

The Swedish National Public Health Survey Health on Equal Terms is a recurring population-based study that investigates health status, health-related behaviours, and living conditions among individuals aged 16 years and older. Initiated in 2004, it is conducted every second year, utilising a stratified random sampling method to ensure national representativeness [19]. The survey is based on items from the Statistics on Income and Living Conditions survey [20], as well as from earlier regional public health surveys that were used before 2004.

Three Swedish government agencies (the Public Health Agency, National Board of Health and Welfare, and Agency for Health Technology Assessment and Assessment of Social Services) together with the Swedish Association of Local Authorities and Regions have sought to develop a common conceptualisation of mental health. This has resulted in a framework in which mental health serves as the overarching construct, comprising both mental well-being and mental ill-health. Mental ill-health is further subdivided into psychological distress and psychiatric conditions [21]. Psychological distress includes emotions that are a common and often normal reaction to life’s challenges, such as unease, worry, anxiety, or stress. Depending on its type and extent, these emotions can affect a person’s ability to manage everyday activities such as work, study, and self-care. Psychiatric conditions include symptoms where the criteria for a psychiatric diagnosis are met and can be divided into psychiatric diseases and syndromes, and neurodevelopmental disorders, respectively. We use this terminology in the current article.

### Study aim

In light of current observations on young people’s mental health, more knowledge is needed about how young people interpret survey questions and relate them to their own health experiences. Such insights not only clarify how survey questions are understood but also offer a deeper understanding of how young people interpret their mental health in today’s societal context. This study aimed to explore young people’s reasoning while interpreting and answering survey items about psychological distress in the Swedish National Public Health Survey.

## Methods

### Mental health in the Swedish National Public Health Survey

Several items in the Swedish National Public Health Survey are used to assess the different dimensions of mental health. Mental wellbeing is assessed using the short version of the Warwick-Edinburgh Mental Wellbeing Scale [22, 23]. Psychological distress is assessed by single-item questions on perceived unease, worry, or anxiety, and on stress (Table 1). Psychiatric conditions are assessed by a single-item question on whether the respondent has ever been diagnosed with depression and by the Kessler-6 scale [24]. In addition, the survey includes questions on suicidal ideation and suicidal attempts, as well as questions on conditions that serve as proxies for mental health status, such as sleep disturbances and perceived general health.

In the present study a qualitative exploratory design was used to explore participants’ experiences and perspectives regarding the survey items about psychological distress. The items have remained unchanged since the survey’s inception, allowing for longitudinal comparisons. Similar items are also included in other international health monitoring instruments, such as the U.S. National Health Interview Survey [25], the Health Survey for England [26], and the World Mental Health Survey [27]. This alignment facilitates cross-national comparisons of public health indicators.

**Table 1:** The two survey items from the Swedish National Public Health Survey and their response alternatives. Translations of items are made by the Public Health Authority

Item	Response alternatives
Do you have any of the following discomforts or symptoms? Anxiety or worry?^1^	□ No □ Yes, minor discomfort □ Yes, severe discomfort
Sw. Har du något eller några av följande besvär eller symtom? Ängslan, oro eller ångest?^2^	
Do you currently feel stressed? By stress, we mean a condition where you feel tense, restless, nervous, uneasy or unable to concentrate.	□ Not at all □ To some extent □ Quite a lot □ Very much
Sw. Känner du dig för närvarande stressad? Med stress menas ett tillstånd då man känner sig spänd, rastlös, nervös, orolig eller okoncentrerad.^3^	

^1^The item is officially translated to English as ”Do you suffer from symptoms of worry and anxiety”, whereas the Swedish wording includes three distinct terms: *ängslan*,* oro och ångest*. In this study, participants reflected on the nuances of the three Swedish concepts

^2^Survey question 11, item h

^3^Survey question 12

### Participants and procedure

Data for this study was generated as part of a project that aimed to explore young people’s views on mental health, including both mental well-being and mental ill-health. The part about mental well-being has been published previously [28]; in this study, we focus on how the participants reason about survey questions related to mental ill-health. The inclusion criterion was being aged 16–25 years. Participants were recruited through social media (Facebook, LinkedIn, and Snapchat) advertisements where information about the study was provided through a link to the project website. Recruitment was initially conducted nationwide and was later targeted to ensure variation between urban and rural areas as well as between men and women. Forty-eight young people declared their interest in participating; 15 of these withdrew. When a reason for withdrawal was stated, it was either a lack of time or ill health. The final sample was a group of 33 participants (22 women, 10 men, and 1 non-binary person) with a mean age of 20.4 years (Table 2). After providing their consent to participate, digital interviews were scheduled via email.

**Table 2:** Participant demographics (*n* = 33)

Characteristic	*n*	%
Gender		
Female	22	67
Male	10	30
Non-binary	1	3
Age		
Range (years)	16–25	
Mean (years)	20.4	
Occupation		
Studying (primary, secondary, or university level)	20	61
Working	10	30
Studying and working	2	6
On sick leave	1	3
Country of birth		
Sweden	30	91
Other	3	9
Region of residence		
Metropolitan area	12	36
Small or medium-sized city	11	33
Rural area	10	30

### Data gathering

Data was gathered via individual interviews based on two of the questions targeting mental ill-health in the 2021 Swedish National Public Health Survey: those about anxiety and stress (Table 1a). These questions were selected due to their relevance to Swedish public health reporting and debate, and because of their general (rather than diagnostic) character. Three interview questions and follow-up questions were prepared for each selected survey question (Table 3). The interview questions were designed by the whole author group, with interviews carried out by ET, HG and SL. This shared analytical responsibility and data gathering enabled critical reflection on emerging interpretations.

**Table 3:** Interview questions: The three interview questions used for each survey question and examples of follow-up questions

Questions during the interview	Examples of follow-up questions
Tell me what you think when you read this question. Which response option do you choose? What are your thoughts as you make that choice?	Question: Unease, worry, or anxiety What might feelings of unease, worry and anxiety mean to you in different situations? What do you understand by “mild problems”? What do you understand by “severe problems”? Question: Stress How do you understand the different response levels in this question? How would you describe stress?

The interview questions were designed to explore the reasoning through which respondents interpreted and answered these survey questions. This process involves question comprehension, information retrieval, judgement and estimation, and finally response reporting [13]. We employed the ‘think-aloud’ approach [29], meaning that participants were asked to verbalise how they were currently reasoning when they read the questions and chose between response options [30]. The interview was guided by the assumption that young people’s experiences and interpretations of mental health questions are situated and context-dependent. Their accounts were viewed as a central source of knowledge and it was not assumed that the researchers’ perspectives provided a more valid understanding than young people’ own interpretations.

Interviews were conducted from February to March 2022 via Zoom and lasted for 24 to 91 min (mean duration 65 min). They were recorded, saved as audio files, and later transcribed verbatim by a professional transcriber.

### Analysis

Transcripts were analysed using inductive qualitative content analysis [31], focusing on the manifest content of the text, i.e. analysing with a low abstraction level and a low interpretation degree [32]. Transcripts were read repeatedly to achieve familiarity with the material and meaning units concerning participants’ interpretations of the survey items were identified and coded. Three of the authors (ET, HG, SL) initially coded one transcript to ensure consistent coding and then each coded an equal number of transcripts. For each survey question, meaning units were identified according to participants’ understanding of the question, how they related it to their own experiences, and their understanding of the response options. For clarity, we analysed each of these areas separately for each question. Meaning units within each of the three areas were assigned codes that was clustered together, capturing similarities and differences in participants’ interpretations and experiences for each item. This resulted in three broad descriptive categories: Understanding the question semantically, Relating the question to one’s experiences, and Interpreting response options. Author MB contributed to discussions throughout the analytic process, including the coding and categorisation of selected transcripts. Differences in interpretation were discussed among the authors until consensus was reached.

## Results

The findings are presented in three sections outlining reasoning relating to each of the two survey questions: (1) how participants semantically understand the question, (2) how they relate their own experiences to the question, and (3) their interpretation of response options. Table 3 provides an analytical overview of the main patterns in the participants’ reasoning, while the nuances and details of these interpretations are presented in the results text below. All names are pseudonyms.

1. UNDERSTANDING THE QUESTION SEMANTICALLY

- Question: Unease, worry, or anxiety

The participants interpreted the first question to include three different levels of the same feeling, even though their perception of the interconnectedness of the levels varied. Some struggled with the meaning of the individual terms. They found them to be close in meaning without being exact synonyms but could not find the words to explain the difference between the terms. Some claimed that the concepts were so different they should be targeted in different questions: when three concepts were included in the same question, the question became confusing and hence difficult to answer.

This could have been a really good question, but if you have anxiety and answer yes, even though you don’t feel unease or worry, then it becomes a strange question for me to answer, because I only have anxiety, not worry or unease. (Isabella, 21 years)

Some, on the other hand, noted a common denominator between the concepts and found it reasonable to make an overall judgement of the three states.

The concept of unease (Sw. ängslan) was tightly connected to worry (Sw. oro) and could be understood as approximately the same state and less serious than anxiety (Sw. ångest). Others perceived the concepts as describing a deteriorating process, but with different interpretations. Specifically, there were variations in the interpretation of whether worry was a more severe state than unease, or the other way around. For some, the underlying feeling of unease could grow into worry in specific situations, which in turn could develop into anxiety. For others, the concept of unease was previously unknown and therefore impossible to comprehend on its own, and some respondents associated unease with a state in animals rather than a human mental state.

This question did not specify any time frame, and participants generally interpreted it as referring to how they usually felt, as an average or summary of their lives.

I’m quite forgetful, so I think I was thinking about what I can remember. But it’s probably more like how I feel in general. I guess it often ends up being about the present, since I’m here right now. But it’s still kind of general. (Ella, 16 years)

Others, however, understood the question as pointing mainly to the present, ranging from the past week to the past month. For some, the lack of a time frame made the question harder to answer, while others felt it gave them more freedom in their recalling of their experiences.

- Question: Stress

Some participants perceived the question about stress as complex and diffuse, whereas others readily recognised what was asked for. The examples included in the question (Table 1) were perceived by some as clear and helpful, and as an indication that stress should be understood as a combination of these states, which to some extent were also perceived as interconnected. Others noted that the examples captured stress to varying degrees, which made it difficult to provide an overall answer to the question. Still others considered it peculiar that so many examples were included, since not all were necessarily associated with stress. In particular, they perceived restlessness, nervousness, and lack of concentration as belonging to emotional states other than stress. Participants tended to understand stress as a more unified experience, rather than as separate and specific states as listed in the examples. When reflecting on the suggested symptoms, they called for the inclusion of physical symptoms in the question, such as stomach pain, which was a recurring experience associated with stress. One suggestion for a more suitable way to capture the occurrence of stress was to ask: ‘Are you able to relax?’

In the stress question, the time frame was specified as ‘currently’ which the participants interpreted in different ways. It could refer to the past days or the past week, as reported by Arvid, 22 years:

I interpret is as a time period of maybe a week, like an average of how it has been.

Others understood it as how they felt at the exact moment of answering. Still others extended ‘at present’ to much longer periods, even up to a year. The participants also noted that focusing too narrowly on the present could give misleading answers if immediate events, such as receiving bad news, overshadowed one’s more general state of mind.

2) RELATING THE QUESTION TO ONE’S OWN EXPERIENCES

- Question: Unease, worry, and anxiety

The participants translated the concepts of unease, worry, and anxiety into experiences of overpowering thoughts, sudden emotional waves, physical pain, and existential reflections. Anxiety could appear abruptly and without cause, whereas worry and unease were often connected to specific situations in school and at work, but also to one’s economic situation, future prospects, and the global climate situation. The three were experienced as different intensities of the same feeling, ranging from transient and manageable states (worry and unease) to a more severe, overwhelming, and unmanageable condition (anxiety).

I mean, I think that unease, worry, and anxiety are basically the same feeling, just at three different levels, and I think unease is maybe the mildest one. So you feel a bit uneasy and so on, but worry is more like when you keep dwelling on something. And anxiety—then you might start thinking less clearly, and that has to do with cortisol and so on… and that’s when it really becomes hard, when the feeling gets really intense and difficult to bear. (Rahma, 19 years)

Unease and worry were described as fleeting and restless thoughts, or as a general sense that something negative was about to happen. Anxiety, by contrast, was experienced as intrusive and escalating thoughts that could ‘burst like a dam’ (Isac, 22 years), often accompanied by feelings of self-doubt and vulnerability. One participant described anxiety primarily through bodily sensations, while unease and worry were experienced as overthinking.

The three concepts could be experienced as waves suddenly washing over a person, sometimes without explanation. Anxiety in particular was experienced as attacking, paralysing, and locking the body, whereas worry and unease were more often tied to concrete situations where it would be natural to experience these feelings, such as exams or job insecurity.

Feelings associated with the three concepts were regarded as part of being human and not necessarily problematic, making it difficult to assess whether or not one’s experience should qualify as a symptom of something pathological. At other times the feelings were understood as normal emotional responses, e.g. feeling anxious when a family member was seriously ill was seen as natural. Unease, worry, and anxiety were also understood to function as a ‘warning light’, signalling that something in life required extra attention. On the other hand, some participants emphasised that they did not struggle with these feelings, explaining that they were simply ‘not the worrying type’ (Rebecca, 24 years), even if they occasionally experienced worry or anxiety.

- Question: Stress

The participants emphasised that stress is experienced differently by different individuals, depending on what one is accustomed to, and therefore the question will be experienced in a variety of ways. For some, the wording of the question itself shaped their understanding of stress compared with how they had defined it beforehand.

With that kind of description, it feels like I’m being stressed more often. I hadn’t previously associated all those things with stress, but I have noticed them more in recent years. (Carl, 23 years)

The participants also pointed out that the question implied that stress is inherently negative, even though in their own lives they recognised positive aspects of stress as well. Stress was regarded as a state that most people enter into at times, as a normal part of life. As long as it remained manageable, it was not seen as a problem. Stress could even be helpful in situations requiring quick completion of tasks, strong performance, or effective problem-solving. It could also encourage productivity and motivation, such as working hard in school. Stress was sometimes experienced as something that could sharpen focus – rather than causing distraction, as suggested by one of the examples in the question – and as preparing the person to face a difficult situation:

Feeling stressed means being productive. I usually don’t get much done unless I’m somewhat stressed, so for me stress is more of a driving force than an enemy. (Rahma, 19 years)

The participants reasoned extensively about the relationship between stress and other states. Anxiety, but also worry, was associated with feelings resembling stress. They noted that worry about uncontrollable matters was linked to the most burdensome forms of stress, and feelings that were perceived as anxiety could in fact be stress. Stress was seen as a state similar to, but less profound than, anxiety. Stress and anxiety could reinforce one another in a kind of vicious circle: stress triggered negative thoughts, which in turn evoked anxiety, which further amplified stress. Like anxiety, stress was also felt in the body. Finally, stress was regarded as resembling anxiety in that both could be tied to specific situations, yet could also arise without any clear cause.

3) INTERPRETING RESPONSE OPTIONS

- Question: Unease, worry, and anxiety

The first question offered three response options. Participants pointed out a clear distinction between minor and severe symptoms, primarily based on the extent to which their everyday life was affected. Severe symptoms were characterised by such a substantial impact on daily functioning that living life as usual was no longer possible, e.g. being unable to leave the house, cancelling social activities, and struggling to manage work or school responsibilities.

Severe discomfort is the kind that makes it hard for me to get through my day. Mild discomfort is something I can still live with. Like, I’m not thinking about it twenty-four seven, maybe only during certain parts of the day, or when someone brings it up. (Nadja, 19 years)

Symptoms were also considered severe when feelings of unease, worry, or anxiety became the predominant emotional state rather than occasional experiences. Symptoms related to anxiety, worry, and unease that would require therapy or medication were also described as severe. In contrast, feelings that were manageable and did not interfere significantly with daily life were classified as mild, and experiencing such emotional states occasionally was regarded as a normal part of life. The response option No could be interpreted as indicating the complete absence of such emotions. However, several participants noted the absence of a response option capturing the experience of having felt unease, worry, or anxiety without perceiving these emotions as problematic:

[I miss some option] between minor problems and no, so that you can feel these things without necessarily being troubled by them. (Carl, 23 years).

Some respondents chose No to reflect this, while others selected Yes – mild symptoms.

- Question: Stress

The question about stress was accompanied by four response options. Participants considered the options to provide sufficient variation, ensuring that all respondents could find an alternative that matched their stress level. However, they also reported difficulties in selecting a single response option that adequately summarised their experience, as the perceived level of stress varied depending on which of the five described states (tense, restless, nervous, worried, or unable to concentrate) they related to most strongly.

I find it difficult because […] you might only feel one of them very strongly, and then those who asked the question won’t know which one you actually mean. (Tanya, 17 years)

Participants also found it challenging to assess how to account for work-related stress, particularly in cases where they experienced high stress levels at work without a corresponding increase in their overall stress in everyday life. The response option Not at all was perceived as unrealistic and misleading, as participants expressed their belief that everyone experiences some level of stress in life, whether positive or negative. Instead, To some extent was regarded as a reasonable choice for individuals with low stress levels. Quite a lot was interpreted as a state of being constantly stressed, such as having a stressful occupation or feeling a persistent pressure to constantly perform at one’s best. Very much was associated with stress levels that significantly interfered with daily life, thereby becoming a serious problem. Some participants suggested that a colour scale or a numerical scale (e.g., from 1 to 10) would better capture the nuances of stress experiences compared with the current response format.

## Discussion

This study sheds light on how young people interpret and answer survey questions about mental health (see Table 4 for a summary). The findings show ambiguities between the ways the questions are formulated and the ways in which our participants understood and responded to them. This, in turn, suggests a risk of both over- and underestimation of symptoms, since similar experiences may be reported differently depending on how respondents understand the wording, their own experiences, the time frame, and the response options. Some participants understood mild variations of stress, unease, worry, and anxiety as part of everyday life, even though the response options No and Yes – mild symptoms could be interpreted differently. Severe symptoms were mostly reserved for experiences they perceived as severely hindering or pathological. Responses also varied depending on whether the question was interpreted as referring to the present moment or a longer period of time, and whether participants answered based on an overall impression or on specific words or examples included in the questions. If questions are perceived as insufficiently specific, variation in responses may reflect not only variation in mental health, but also variation in how the questions are interpreted.

**Table 4:** Summary of patterns in young people’s reasoning (see Result text for detailed interpretations)

Dimension of reasoning	Question: Unease, worry, or anxiety	Question: Stress
Understanding the question semantically	The three terms were understood as related but not identical; some saw them as different intensities of the same feeling, others found them too distinct to be combined in one item. The lack of a time frame was usually interpreted as a broad time frame, though some narrowly focused on the present moment.	The question was perceived either as clear or as diffuse. The examples helped some respondents but confused others, particularly when listed states were not seen as clearly related to stress. ‘Currently’ was interpreted inconsistently, from the immediate present to up to a year earlier.
Relating the question to own experiences	Experiences ranged from fleeting thoughts and situational worry to overwhelming anxiety, often linked to school, work, finances, and the future. Participants often described the concepts as different intensities of a shared emotional experience.	Stress was described as both negative and positive, sometimes motivating and sharpening focus. It was seen as related to, but distinct from, anxiety and worry, and as something shaped by context and individual tolerance.
Interpretation of response options	Clear distinction between mild and severe symptoms, mainly based on impact on daily life. Mild symptoms were often seen as normal experiences and should not be considered as anything pathological. Several participants missed an option for having these experiences without considering them problematic.	Response options offered variation, but respondents found it hard to choose one answer when stress varied across the listed examples or across settings such as work versus everyday life. ‘Not at all’ was often seen as unrealistic.
*Ambiguities shown in the analysis*	*Whether the three concepts refer to the same feeling or distinct emotional states; whether the item captures normal human states vs. symptoms of ill-health; whether the absence of a time frame encourages a general or present moment-oriented answer*	*Whether the question solely reflects stress as a negative phenomenon; whether the listed examples reflect stress or other states; and whether “currently” refers to the present moment, recent days, or a longer period.*

A key challenge in measuring mental health is that it spans a continuum from common, natural emotional states and reactions to more severe conditions involving psychiatric symptoms. That challenge was highlighted by our participants. While answering the questions, the participants seemed to make sense of the survey questions based on their interpretations about what constitutes ordinary life experiences in contrast to serious mental health problems, a finding that is not new among Swedish young people [14]. Serious mental health problems were interpreted as emotional states that were interfering with their daily life, and for such situations, they used response options such as ‘severe discomfort’ or ‘very much’. In contrast, when interpreting unease, worry and anxiety as a natural part of life, their reasoning showed that they could choose Yes – mild symptoms, but without defining it as ill-health, whereas others could answer No for the same feelings. This ambiguity makes the distinction between the response options unclear.

Also, the presence of ‘stress’ was not necessarily seen as a negative state. Similar patterns have been observed among university students in the UK, who framed stress as a normal feature of everyday student life, helping them to manage demands and navigate the transition into adult life [33]. This normalised view of stress may contrast with the survey’s intended use of response options as graded indicators of symptom intensity, and points to a potential mismatch between how response options are interpreted by respondents and how they are intended to be used in population health monitoring. According to our findings, experiencing the symptoms asked about in the survey does not necessarily imply they are experienced as ill-health. Similar findings have been reported in previous research on younger adolescents’ (15 years) meaning-making of survey questions in their everyday lives [14]. Our study extends this insight to a slightly older age group (16–25) and describes young people’s reasoning in the response situation.

Changes in how young people interpret mental health concepts could be understood in relation to broader societal developments, including increased public education, anti-stigma efforts, and more narratives in the press and social media about mental ill-health [34]. Initiatives by organisations such as the World Health Organization, as well as national campaigns like Mind in England, Beyond Blue in Australia, and Hjärnkoll in Sweden, have contributed to more widespread and nuanced understandings of mental health. These developments may help explain why the survey items in this study were interpreted in relation to contemporary expectations and meanings, which are likely to differ from those at the time the survey was introduced in 2004. However, to date, it remains unclear how conceptualisations of mental health have changed over time or how such changes may affect reporting practices [35, 36]. Further research is required to enhance our understanding of these issues.

Our findings resonate with conceptualisations of the survey responding as a process involving comprehension, retrieval, judgement, and response mapping [13]. Comprehension involves interpreting the intended meaning of survey items in relation to everyday language [37]. Difficulties in semantic comprehension were evident when participants struggled to disentangle closely related concepts such as ‘unease, worry, and anxiety,’ echoing earlier observations that respondents often face problems when everyday terms are used in precise survey contexts [37]. Also, the variation in how temporal concepts such as ‘currently’ were interpreted indicates that the term has no clear temporal meaning. This may affect respondents’ retrieval of experiences, as retrieval depends on which time frame is activated when respondents search their memory [13]. We therefore suggest that a more clearly defined timeframe may contribute to a clearer understanding of what is being asked about. Comparing these survey questions with diagnostic psychiatric manuals (e.g. DSM-5, ICD-10), the psychiatric manuals do include time frames.

Our findings indicate that the survey questions are understood within a societal discourse that frames experiences such as anxiety and stress as both normal and sometimes productive aspects of everyday life. Previous studies also highlight young people’s normalisation of mental health issues, and that young people (15–18 years) regard mental health problems as common in their generation, attributing this development partly to reduced stigma, greater openness, and better access to help compared to previous generations [18]. This raises the possibility that unchanged survey questions may not capture the same underlying phenomena today as they did two decades ago. Qualitative studies on children and adolescents have concluded that there is a need for a shared language on mental health, because there is much uncertainty surrounding mental health concepts and how to apply them, e.g. how to make the distinction between everyday challenges and normal ups and downs compared to mental health problems [38]. This uncertainty has also been demonstrated in qualitative studies showing that children and adolescents recognise mental health terminology but struggle with its application, reflecting changing and sometimes conflicting societal discourses surrounding mental health [39]. The normalisation suggested in our study may contribute to this uncertainty by blurring the boundary between ordinary life experiences and mental health problems. This was reflected in the participants’ reasoning when they distinguished between experiences that were perceived as ‘normal’ or situational, and those that interfered with daily functioning, therefore motivating more severe response options. This distinction is consistent with Eriksson and Stattin’s differentiation between a stable poorest mental health profile and a growing profile characterised by high psychosomatic symptoms but average perceived overall health [5]. Our findings offer a possible response-process perspective on this pattern, as the main ambiguity for our participants appeared to be related to the responses No and Yes – mild symptoms, rather than in identifying severe mental ill-health. This suggests that our participants actively reflected on what should count as a mental health problem and how their experiences should be represented in the response options.

However, the normalised use of mental health terminology may make psychological distress easier to articulate but may also contribute to a medicalising discourse in which ordinary experiences are interpreted as symptoms of distress [17, 40]. Young people may appropriate and transform such terms rather than use them in accordance with their clinical meanings [40]. This should also be noted when interpreting our findings.

If the understanding of survey terms and the phenomena they aim to measure change over time, using static items during long time periods may compromise survey reliability and validity. Our findings illustrate this risk. Our participants interpreted the central concepts in the items in relation to contemporary understandings of everyday experiences. For instance, ‘stress’ seemed to be accepted as an integrated aspect of everyday life, a view that might be more normalised today compared to when the survey was launched in 2004. This interpretation is also consistent with our related qualitative findings showing that young people describe experiences in everyday life as shaped by their ability to use their resources and strategies to navigate stressors [28]. Although research as early as the 1950s, particularly by Selye [41], sought to promote a broader understanding of stress by including both negative and positive dimensions, this perspective may not have become established in young people’s general view of stress until more recently.

However, the societal view on stress has undergone a shift, from being framed more narrowly as physiological and negative responses to challenges to being understood as a natural response to challenges that people experience and manage in everyday situations [42, 43]. The wide span of interpretation that the concept of stress includes was visible in the formulation of the stress question in our study: it was listed with five examples, narrowing the participants’ understanding of the phenomenon. This was noted by the participants in our study, who claimed that the formulation provided them with new insights into the meaning of stress.

### Methodological considerations

Trustworthiness in the analytical process was ensured by having three analysts (ET, HG, SL) code the data set and engaging in recurring discussions about theme generation with a fourth analyst (MB). The author group had expertise in mental and public health, as well as experience of qualitative interviewing and analysis concerning mental health in various population groups. This formed our pre-understanding and the meanings we attended to and how we interpreted participants’ accounts. We regarded the participants as knowledgeable about their own experiences and their accounts as valuable for understanding how the survey questions were interpreted.

Even though transferability of the findings to young adults more broadly may be limited by the characteristics of the study sample and the Swedish setting, ‘think-aloud’ designs are intended to produce in-depth understanding of how survey questions are interpreted and answered by respondents [29]. Our study provides insights into the participants’ experiences and perspectives, which may be relevant to other individuals and contexts with similar characteristics. It is likely that the participants who chose to take part had a particular interest in mental health issues, however the sample displayed variation across key characteristics such as age, gender, educational and work situation, and place of residence. This heterogeneity strengthens the analytical relevance of the findings and supports their transferability to other survey settings in which young people are asked to interpret and respond to similar self-report questions about mental health [44, 45].

### Implications for public health practice

Our findings suggest that, in relation to the terms ‘unease’, ‘worry’, and ‘anxiety’, unease (Sw. ängslan) was the least clearly understood and least frequently used term among participants. This uncertainty contributed to difficulties in interpreting the question and in distinguishing between response options. In contrast, anxiety was consistently described as the most serious state. This pattern indicates that our respondents did not treat unease, worry, and anxiety as three separate types of experiences, but rather as closely related terms that differ mainly in their perceived intensity. This raises the question of whether the inclusion of ‘unease’ adds meaningful information or instead introduces ambiguity that may affect how respondents interpret the question as a whole. A reduced number of concepts in the item may preserve the intended distinction while reducing respondent confusion.

Further suggestions for improvement include developing questions that more clearly capture how the emotional states referred to affect respondents’ everyday life. This may help respondents distinguish between what they consider normal feelings and states they perceive as more burdensome or indicative of ill-health, consistent with diagnostic psychiatric manuals, which cover how symptoms interfere with everyday functioning as well as time frames.

Including a defined time frame would probably improve the consistency in young people’s responses, as the participants used very different temporal reference points when answering the same question.

Looking ahead, it is possible that this generation will carry its current understanding of mental health problems into later life, which would in turn reproduce the same dilemma in comparisons across measurements that we currently observe when comparing the responses of young adults with those of earlier cohorts. To better understand this, a corresponding ‘think-aloud’ study could be conducted among, for example, today’s 35–45-year-olds and then repeated when the current 15–25-year-old cohort reaches that age.

In addition, participatory approaches could support the development of shared and meaningful language in mental health surveys by involving young people in the formulation and testing of questions and response options.

## Conclusion

This study shows that young people’s reasoning when answering survey questions on mental health is shaped by cognitive processes through which everyday experiences, general perceptions, and personal interpretations of symptom severity and time frame are active in the response situation. The results suggest that survey items assumed to be stable over time may, in fact, measure different underlying constructs and phenomena across different time periods. The lack of time frames and a clear link to decreased daily functioning introduced uncertainty in how to interpret the questions. Interpretation should not be regarded as a flaw in subjective inquiry, but as an inherent condition of language-based communication. The challenge for population surveys is instead to identify and reduce unintended variation in interpretation so that responses can support valid comparisons and conclusions.

These findings highlight the importance of interpretive awareness when drawing conclusions from self-reported mental health data among young people from public health surveys, as our findings show the risk of both over- and underestimation of mental health problems. It seems urgent to combine surveys with qualitative approaches to gain a comprehensive view of mental health experiences and how young people today make sense of them.

## Data Availability

The dataset analysed during the current study is available from the corresponding author on reasonable request.
